# Supplementary material for: Availability, cost and affordability of essential cardiovascular disease medicines in the south west region of Cameroon: Preliminary findings from the Cameroon science for disease study
Source: PLoS One. 2020 Mar 4;15(3):e0229307. doi: 10.1371/journal.pone.0229307 (PMC7055918; doi:10.1371/journal.pone.0229307)
Supplement: S1 File — (DOCX) [file pone.0229307.s001.docx]

**CAMSCID 1: A Situational Analysis**

**Questionnaire**

**Date: ____________________________________ Survey area number: ________________________**

**Name of Town/village/district: ____________________________________________________________**

**Name of medicine outlet: ________________________________________________________________**

**Name of medicine outlet unique survey ID: __________________________________________________**

**Type of medicine outlet:**

**Name and contact number of Manager of outlet: _____________________________________________**

**Name(s) and contact number(s) of persons who provided information (if different from the manager):_____________________________________________________________________________**

| **SECTION 1 : CHARACTERISTICS OF HEALTH FACILITY** | | | |
| --- | --- | --- | --- |
| NAME OF HEALTH FACILITY: _______________________________________________________________  Contact of Health Facility 1) \|__\|__\|__\|__\|__\|__\|\|__\|__\|__\| 2) \|__\|__\|__\|__\|__\|__\|\|__\|__\|__\| | | | |
| 001 | In which region is the health facility located?  1=Adamaoua, 2= Facility, 3=East, 4=Littoral, 5=North, 6= Far North, 7= West, 8 = North West, 9= South West, 10=South | | \|__\| |
| 003 | What is the category of your health facility?  1= Urban, 2= Semi-urban, 3=Rural | | I ___I |
| 005 | What is the average population served by your health facility? | I ___I I ___I I ___I I ___I I ___I I ___I | |
| 007 | What is the n° of health workers in the health facility? | I ___I I ___I I ___I | |
| 008 | What is the n° of nurses in the health facility? | I ___I I ___I I ___I | |
| 009 | What is the n° of doctors (GPs) in the health facility? | I ___I I ___I I ___I | |
| 010 | What is the total n° of specialists in the health facility? | I ___I I ___I | |
| 011 | What is the n° of specialists taking care of CVDs (internal medicine or cardiologists) in the health facility? | I ___I I ___I | |
| 012 | What is the total staff in the health facility (including non-health workers e.g cooks, etc…)? | I ___I I ___I I ___I | |

| S**ECTION 2: AVAILIBILITY OF FREQUENTLY USED CARDIOVASCULAR** DRUGS | | | |
| --- | --- | --- | --- |
| 401 | Does the centre have a list of essential medicines? | 1=Yes, 2= No | \|__\| |
| 402 | If yes, specify: a) WHO b) other |  | |
| 402 | If yes, is a copy available (Tick yes only if the survey officer can see a copy)? | 1=Yes, 2= No | \|__\| |
| 403 | Thiazide diuretics? | 1=Yes, 2= No | \|__\| |
| 404 | If yes, circle which one: 1=hydrochlorothiazide, 2= Indapamide, 3= chlortalidone | | |
| 405 | Furosemide used for treatment of hypertension? | 1=Yes, 2= No | \|__\| |
| 406 | Calcium channel blockers (CCB)? | 1=Yes, 2= No | \|__\| |
| 407 | If yes, circle which one: 1=Amlodipine, 2= Nifedipine 10 mg, 3= Nifedipine 20 mg, 4= other CCB | | |
| 408 | Angiotensin converting enzyme inhibitor (ACEI)? | 1=Yes, 2= No | \|__\| |
| 409 | If yes, circle which one: 1=captopril, 2= ramipril, 3= perindopril, 4=lisinopril, 5=other ACEI | | |
| 410 | Angiotensin receptor blockers (ARBs)? | 1=Yes, 2= No | \|__\| |
| 411 | If yes, indicate which one: 1=valsartan, 2= losartan, 3= candesartan, 4= other ARB | | \|__\| |
| 412 | Combination treatment for hypertension? | 1=Yes, 2= No | \|__\| |
| 413 | If yes, circle which one: 1= CCB + Thiazides, 2= ACEI + Thiazides, ACEI , 3= CCB + ACEI or ARBs, 4= other | | |
| 414 | Beta blockers? | 1=Yes, 2= No | \|__\| |
| 415 | If yes, circle which one: 1=propanolol, 2= atenolol, 3= beta blocker with vasodilator action (Nebiv/carvedi/metopr/celiprolol)l, 4=other BB | | |
| 416 | Spironolactone? | 1=Yes, 2= No | \|__\| |
| 417 | Amiloride? | 1=Yes, 2= No | \|__\| |
| 418 | Aldomet? | 1=Yes, 2= No | \|__\| |
| 419 | Metformin? | 1=Yes, 2= No | \|__\| |
| 420 | Sulfamide like? E. g glibenclamide or glicazide or else | 1=Yes, 2= No | \|__\| |
| 421 | Insulin? | 1=Yes, 2= No | \|__\| |
| 422 | Statin? E. g simvastatin, atorvastatin, or else | 1=Yes, 2= No | \|__\| |
| 423 | Aspirin | 1=Yes, 2= No | \|__\| |
| 424 | Long acting penicillin | 1=Yes, 2= No | \|__\| |
| 425 | Carbamazepine | 1=Yes, 2= No | \|__\| |
| 426 | Phenytoin | 1=Yes, 2= No | \|__\| |
| 427 | Valproic acid | 1=Yes, 2= No | \|__\| |
| 428 | Phenobarbital | 1=Yes, 2= No | \|__\| |

| **SECTION 3: WHO ESSENTIAL LIST; AVAILABILITY AND** **COST** | | | | | | | |
| --- | --- | --- | --- | --- | --- | --- | --- |
|  | A | B |  | C | D | E | F |
|  | Is there a list of essential medicines? | Yes = 1, No = 2 | [ ] |  |  |  |  |
|  | If yes, is a copy available? | Yes = 1 ( only if the survey officer can see a copy), No = 2 | [ ] |  |  |  |  |
|  | Generic name, dosage form, strength | Available; yes = 1, No = 2 | [ ] | Pack size found | Price of pack | Unit price | Comments |
|  | Acetylsalicylic acid  100 tab | Yes = 1, No = 2 | [ ] |  |  |  |  |
|  | Amlodipine  5mg tab | Yes = 1, No = 2 | [ ] |  |  |  |  |
|  | Atenolol  50 mg tab | Yes = 1, No = 2 | [ ] |  |  |  |  |
|  | Captopril  25mg tab | Yes = 1, No = 2 | [ ] |  |  |  |  |
|  | Digoxin  0.0625mg | Yes =1, No =2 | [ ] |  |  |  |  |
|  | Furosemide  20 mg tab | Yes =1, No =2 | [ ] |  |  |  |  |
|  | Glyceryl trinitrate  0.3 mg tab | Yes =1, No =2 | [ ] |  |  |  |  |
|  | Hydrochlorothiazide  25 mg tab | Yes =1, No =2 | [ ] |  |  |  |  |
|  | Methyl dopa  250mg tab | Yes =1, No =2 | [ ] |  |  |  |  |
|  | Nifedipine  10 mg tab | Yes =1, No =2 | [ ] |  |  |  |  |
|  | Simvastatin  20 mg tab | Yes =1, No =2 | [ ] |  |  |  |  |
|  | Spironolactone  50 mg tab | Yes =1, No =2 | [ ] |  |  |  |  |
