# Supplementary material for: Availability, cost and affordability of essential cardiovascular disease medicines in the south west region of Cameroon: Preliminary findings from the Cameroon science for disease study
Source: PLoS One. 2020 Mar 4;15(3):e0229307. doi: 10.1371/journal.pone.0229307 (PMC7055918; doi:10.1371/journal.pone.0229307)
Supplement: Attachment — Submitted filename: PLOS comments.docx [file pone.0229307.s002.docx]

Availability, Cost and Affordability of Essential Cardiovascular Disease Medicines in the South West Region of Cameroon: Preliminary Findings from the Cameroon Science for Disease study

Reviewer Comments

**Introduction;**

**Line 47.** CVD is the leading cause of death globally. ……

**Line 49/50.** Recent projections show that suggest that deaths from CVDs will continue to rise, surpassing all other causes by 2030.

I thought these two lines from the start are contradictory. Because if the first says CVD is the leading cause of death globally, and then the next says it will surpass all other causes by 2030, then it needs to be clarified.

**Methodology**

**Line 99.** I think it would be important to throw in some line that explains what a health district (HD) and how it is structured, because this concept is used in other countries but it actually mean varied things. OR even probably explain aliitle elaborately how the Cameroon health system is structured and the medicine distribution and supply chain. Do people access medicines from public and private facilities at a cost?

**Line 108/109**. What was the justification for choosing the main public hospital, AND then four others closest to it? I think a justification line needs to be added in there, because I note that in line 112/113, the community Pharmacies closest are included and their inclusion was justified. But I am wondering what that also implies for introducing clustering biases an effects in the data.

**Line 118**, implies to me that the sampling and selection of outlets especially community pharmacies, was based more on convenient sampling probably to reduce study costs. But I am wondering whether this could have had recognizable effects on the data an ultimately the understanding and interpretation of results?

**Line 171/174.** I thought this section touches on ethics and data confidentiality issues and therefore could have fitted well in the section of ethics at the start of the methodology section. But again this depends on how PLOS wants a paper structured.

**Results**

**Line 176/181**. I thought the write-up in this section was sufficient and in any case could be expanded, making the Table 1, irrelevant because its information has already been explained. I find it not adding any value to this.

**Line 195 onwards**. I find that the write-ups are virtually a repetition of the table 2. It is always recommended that once you use a table, you could only explain the key issues in the table that the readers should take note of. But you cannot repeat an entire table by writing.

**Line 236.** I should have made this comment in methodology. The study considered the wage of a lowest paid unskilled government worker. Can they include the justification for using this reference category? Is it that the lowest paid government workers are the ones who have a higher burden of disease in this CVD or NCD area? Or is it that once the lowest paid Government worker can afford this treatment then everyone else above them is expected to afford? How does a lowest paid government worker compare with a peasant individual without any gainful work or in agriculture? Would it have been useful to compare with incomes of those in agriculture or no gainful work at all? I think some clarification for selection of the reference case may be convincingly necessary.

**Line 280.** I found Table 4, not necessary. In the first place I don’t know what the figures are, and whether they are dollar values or something else or percentages. I would recommend that Table 5 communicates better the idea of cost and affordability. This is because cost, moving forward is quoted in dosage (30 days of required dosage). So I would think Table 4 is generally irrelevant.

**General**

**These drugs seem to have been taken as a package. However, I was wondering for CVD for example, whether an entire drug package should be available to treat a CVD case OR there is this one or two essential drug that if not available, then a patient is in problems. Could you have identified that “essential” of “essentials” and assessed their availability?

*****I would have expected some result on the possible reasons or factors associated with the variation in MPs across the sectors, and this would tie in well with an earlier comment I made of …describing the structure of the health system and probably the supply chain so we can know where the costs and therefore higher markups are likely to be associated.

***We could have also benefitted from knowing whether geographical considerations have an influence on costs of drugs, that’s looking at urban and rural, those within vicinities of big public facilities compared to those a little further away from them. How do their medicine prices compare? I know you did this comparison only on ‘availability’.

**Discussion**

**Why was aspiring missing in virtually public facilities? Does it have something to do with essential medicines list? Or change in prescriptive policy? I thought I would find this argument in the discussion.

**Line 307/313**. I would have thought some small amount of qualitative would have explained these facts you are imputing on doctors not prescribing certain drugs. I think a KI interview would have added value.

**Line 350.** I think the issue of a reference wage case, needs to be explained, otherwise it may end-up in this section of limitations. But it should be elaborated in the earlier sections.

**** I thought results would have also been contextualized alittle more. Availability was very low in public facilities. How does this relate with the broader context of the Cameroon economy, public expenditure on health, per capital health expenditure, or generally the health financing structure of Cameroon, or the generic problems that are often reported of medicine distribution and supply chain challenges, pilferage (medicine thefts from public facilities ending into private), the big numbers that report in public facilities and hence depleting the available stocks, medicine waste, etc. These issues are not unique, and so I am surprised that they seem not to feature prominently in the discussion.

***I did ask a question, whether medicines in public facilities were also paid for. And this comment could be because you never explained to us the structure of Cameroon health system. I know many public health systems are free, and so I could not understand what cost mean for a public system that may be free being costed.

***Some comments about if there exists a policy reform now on NCDs in Cameroon may be good to understand whether NCD agenda has now been put on the priority list of issues. Some health systems are still focused on CDs and even their EMS has not changed to reflect the new NCD realities. Could this have an impact on the low stocks we have witnessed in the public and the confessional sectors?
